# Analysis of Microbial Community Structure and Diversity in Different Soil Use Types in the Luo River Basin

**DOI:** 10.3390/microorganisms13092173

**Published:** 2025-09-17

**Authors:** Li Dai, Xiaolong Hao, Tong Niu, Zhen Liu, Yanmei Wang, Xiaodong Geng, Qifei Cai, Juan Wang, Yongyu Ren, Fangming Liu, Hongen Liu, Zhi Li

**Affiliations:** 1 College of Forestry, Henan Agricultural University, Zhengzhou 450046, China; 2 National Forestry and Grassland Administration Key Laboratory for Central Plains Forest Resources Cultivation, Henan Agricultural University, Zhengzhou 450046, China; 3 Henan Province Engineering Technology Research Center for *Idesia*, Zhengzhou 450046, China; 4 Resources and Environment College, Henan Agricultural University, Zhengzhou 450046, China; 5 Key Laboratory of Farmland Quality Conservation in Huang-Huai-Hai Plain, Ministry of Agriculture and Rural Affairs, Zhengzhou 450046, China; 6 Key Laboratory of Soil Pollution Prevention, Control and Remediation in Henan Province, Zhengzhou 450046, China

**Keywords:** Luo River basin, land type, soil bacteria, high-throughput sequencing, redundancy analysis

## Abstract

The Luohe River boasts a profound historical heritage. Due to long-term impacts of human activities along its banks, significant variations in soil environmental conditions may exist across different land use types within the region. This study focused on four land use types (farmland, bamboo forest, grassland, and abandoned land) in Luoning County of the Luohe River Basin and employed high-throughput sequencing technology to analyze the characteristics of soil microbial communities and differences in soil nutrients. The results showed the following: There were significant differences in soil nutrients and microbial diversity among different land use types. Specifically, the organic matter content in farmland was significantly higher than that in bamboo forests (*p* < 0.05), and the available phosphorus content in farmland was significantly higher than that in abandoned land (*p* < 0.05); the abandoned land had a significant advantage in alkali-hydrolyzable nitrogen and available potassium contents (*p* < 0.05) but the lowest soil water content (*p* < 0.05). Microbial diversity indices indicated that Pielou’s evenness index (Pieloue) in farmland was significantly higher than that in grassland. The bacterial community was dominated by Acidobacteria, Proteobacteria, and Actinobacteria. At the genus level, available potassium was the key factor affecting the top 20 dominant bacterial genera. Redundancy Analysis (RDA) showed that pH was the core environmental variable driving the variation of bacterial community structure. Metabolic pathway analysis revealed that biosynthetic metabolism was the main pathway, and grassland exhibited outstanding performance in the secondary metabolite synthesis pathway. The results of this study fill the gap in soil microbial ecology research in this region and provide a theoretical basis for the sustainable utilization of land resources and agricultural ecological management in the Luohe River Basin.

## 1. Introduction

Soil constitutes one of the Earth’s key carbon reservoirs, with its organic carbon accumulation exerting significant influence on global climate change and soil nutrient cycling [1,2,3]. As the primary medium for plant and microbial growth, soil provides nutrients, moisture and habitat for organisms, forming the foundation that sustains the structure and function of terrestrial ecosystems [4,5]. Within soil ecosystems, microorganisms serve as pivotal biological components, participating in carbon and nitrogen cycles alongside organic matter transformation. They exert direct or indirect influence upon plant development and soil health; for instance, the phylum Acidobacteria promotes the cycling and transformation of soil structure and nutrients [6,7,8,9,10,11,12,13]. Soil microorganisms are highly sensitive to environmental changes [14]. Their community structure and diversity serve as effective indicators of soil quality and ecological stability [15,16], and they also play a significant role in soil and water conservation [17]. Reasonable management measures can optimize microbial community structure by regulating environmental factors, thereby promoting vegetation restoration and improving soil physicochemical properties [18,19]. The two mutually influence and reinforce each other. For instance, the formation of soil aggregates is directly related to microbial organic matter binding, fungal hyphae wrapping around particles, and the mineralization of organic carbon [20]. Conversely, soil aggregates enhance the stability and integrity of soil structure, thereby providing an optimal habitat for microorganisms [21,22]. However, different land use types exhibit soil environmental conditions of [23], which significantly influence microbial community structure and functional expression [24,25]. Soil bacteria, as a significant component of microbial communities, constitute 70–90% of the total microbial biomass. Changes in their community structure serve as important indicators of soil health status [26,27,28]. Liu et al. [29] analyzed different land use types along the Jialing River riparian zone, finding that establishing wetland ecosystems in this area would promote soil biodiversity and stability. Hu et al. [30] compared bacterial community structures in woodlands and paddy fields, discovering that woodland bacteria may exhibit stronger microbial interactions.

The Luo River possesses profound historical significance, historically revered as a sacred river. As a highly prominent tributary of the middle and lower reaches of the Yellow River, its cultural value and historical stature are comparable to that of other Yellow River tributaries such as the Wei River and the Fen River [31]. The Luo River is the largest primary tributary within the Yellow River basin (Henan section). Due to prolonged human activity along its banks, land use patterns within the basin exhibit distinct spatial distribution characteristics [32]. Due to the region’s unique geographical characteristics, soil environmental conditions may exhibit significant variation across different land use types within the area. However, current research on the region’s soil ecosystems has predominantly focused on mineral resources, with a notable absence of studies systematically examining the impact of land use practices on soil quality and health from a microbial ecology perspective. In light of this, the present study focuses on the Luo River basin within Luoning County, Henan Province, examining four typical land use types: abandoned farmland, cultivated fields, grassland, and bamboo forests. By integrating high-throughput sequencing technology with analyses of soil physicochemical properties, it systematically compares the structure and diversity of soil bacterial communities across different land use regimes, alongside their coupling relationships with key environmental factors. This study incorporates microbial community indicators into the land ecological assessment framework of the Luo River basin, aiming to elucidate the mechanisms by which land use types shape soil microbial ecosystems under anthropogenic disturbance. It addresses gaps in soil microbial ecology research within this region, with findings providing scientific evidence and theoretical underpinnings for rational land resource utilization and enhanced ecological functionality.

## 2. Materials and Methods

### 2.1. Overview of the Study Area

**Figure 1:**
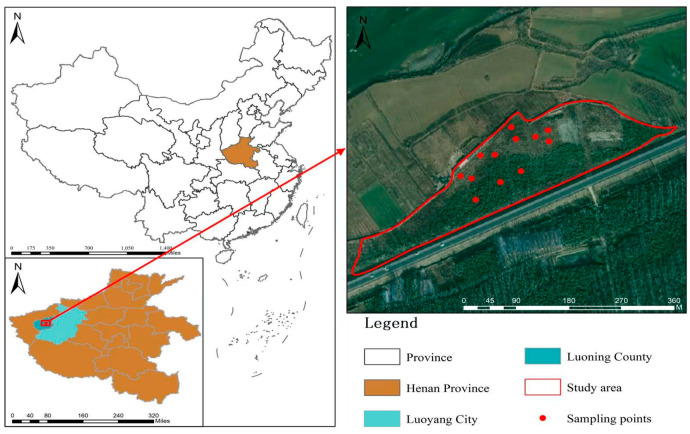
Location schematic diagram of sampling points.

The study site is situated within the Luo River basin in Luoning County, Henan Province (between 34°05′ and 34°38′ north latitude, 111°08′ and 111°49′ east longitude). Luoning County experiences a warm temperate continental monsoon climate with four distinct seasons, featuring a frost-free period of 216 days annually. with an annual mean temperature of 13.7 °C and annual precipitation ranging between 600–800 mm. The topography exhibits a distinctive ‘seven parts mountain, two parts plateau, one part river valley’ configuration, fostering diverse ecosystem types and rich biodiversity [33]. To investigate the soil microbial communities and physicochemical properties across different land use types within Luoning County of the Luo River basin, four distinct land use categories were selected for analysis: agricultural land (NGD), grassland (CD), abandoned land (LHD), and bamboo forest (ZL) (Figure 1).

### 2.2. Soil Sample Collection and Preservation

Soil samples were collected in March 2024, following this specific procedure: surface debris was cleared to expose the soil surface; using a 2.5 cm diameter soil auger, three sampling points were established at each plot to collect soil from a depth of 0–30 cm. Upon completion, the collected soil samples were promptly placed into sterile self-sealing bags and stored in dry ice for low-temperature preservation. These samples were subsequently utilized for high-throughput sequencing of soil microorganisms and determination of soil physicochemical properties.

### 2.3. Determination of Soil Physical and Chemical Properties

The methods for determining soil physicochemical properties are as follows [34]: Soil pH was measured using a pH meter after shaking a soil/water suspension (1:2.5) for 30 min. Soil water content (SWC) was determined using the oven-drying and weighing method. Soil bulk density (SBD) was measured using the ring knife method. Soil organic matter (SOM) was determined by the potassium dichromate oxidation-external heating method. Alkali-hydrolyzable nitrogen (AN) was determined by the alkali-hydrolysis diffusion method. Available phosphorus (AP) was measured using the NaHCO_3_ molybdenum antimony colorimetric method. Available potassium (AK) was analyzed by NH_4_OAc extraction followed by flame photometry.

### 2.4. DNA Extraction and PCR Amplification

Upon removal of samples from the refrigerator (with three replicates per plot, totaling 12 samples), precisely weigh 0.2–0.5 g of each sample. Place these into centrifuge tubes containing extraction lysis buffer and subject them to grinding. For the pre-treated samples, nucleic acids were extracted using the MagBeads Fast DNA Kit for Soil (MP Biomedicals, Irvine, CA, USA) [35]. The extracted DNA was subjected to 0.8% agarose gel electrophoresis for molecular size determination, followed by quantification using Nanodrop. PCR amplification of microbial community DNA from soil samples was performed using universal primers 338F (5′-ACTCCTACGGGAGGCAGCA-3′) and 806R (5′-GGACTACHVGGGTWTCTAAT-3′), targeting the V3–V4 variable region of the bacterial 16S rRNA gene. The reaction system was pre-denatured at 98 °C for 5 min, followed by 28 cycles of amplification (98 °C for 30 s, 55 °C for 45 s, 72 °C for 45 s), with a final extension at 72 °C for 5 min. The product was then stored at 12 °C. The amplified products were verified by 2% agarose gel electrophoresis. Following detection, the target fragment was excised and recovered using the Axygen Gel Recovery Kit (New York, NY, USA). Subsequently, libraries were prepared using Illumina’s (San Diego, CA, USA) TruSeq Nano DNA LT Library Prep Kit. Sequencing was performed using the NovaSeq 6000(San Diego, CA, USA) SP Reagent Kit (500 cycles) for 2 × 250 bp paired-end reads (indicating the read length). The sequencing work was outsourced to Shanghai Paisenuo Biotechnology Co., Ltd. (Shanghai, China).

### 2.5. Data Processing and Analysis

Using QIIME2(2022.11) [36] tools, the workflow involves importing raw fastq files into a format processable by QIIME2, followed by modification and refinement to analyze microbiome biological information. Raw sequence data undergoes decoding via the demux plugin, followed by primer removal using the cutadapt(2.3) plugin. Subsequently, the DADA2(QIIME2 2022.11) [37] plugin performs quality filtering, denoising, assembly, and chimera removal (with an expected error rate of 2% for both forward and reverse reads, and a trimming length of 223 and 230 bases, respectively). The sequences obtained above were merged based on 100% sequence similarity, generating feature sequences (ASVs) and abundance data tables. By employing the SILVA_138_1 99% database, 16S rRNA sequences were aligned against reference sequences within the database to obtain taxonomic information corresponding to each 16S rRNA. QIIME2 software (Version 2022.11) was utilized to visualize the compositional distribution of samples at the phylum and genus levels, presenting microbial community structural characteristics through relative abundance. Alpha diversity analysis was conducted using QIIME2 software, R (version 3.6.1) with the ggplot2 package. Principal coordinate analysis (PcoA) was performed using QIIME2 software, R (version 3.6.1) with the ape package. LEFSe analysis was executed using Python’s LEfSe package, R (version 3.6.1) with the ggtree package, and other relevant tools. Concurrently, functional potential of soil microbial communities was predicted using PICRUSt2 software and R (version 3.6.1) based on ASVs derived from 16S rRNA sequencing and annotation against the SILVA_138_1 database. Correlation analysis methods were employed to investigate associations between microbial communities and relevant nutrient factors. Visualization of relationships between variables was achieved through Redundancy Analysis (RDA) plots and correlation heatmaps. Visualizing relationships between variables (The closed-reference clustering method was used for the PICRUSt analysis). Basic data organization was performed using Microsoft Excel 2019 software. Statistical analysis of soil physicochemical properties, alpha diversity indices, and metabolic pathways was conducted using Origin 2024 and IBM SPSS 26.0 software, including significance and Spearman correlation tests, with corresponding graphical representations produced. In the text, A denotes *p* > 0.05, B denotes *p* < 0.05, C denotes *p* < 0.01, and D denotes *p* < 0.001.

## 3. Results

### 3.1. Analysis of Soil Physical and Chemical Properties of Different Land Use Types

Through the analysis of soil physicochemical properties under different land use types, Table 1 reveals distinct variation patterns in soil indicators among the various land use types. Cultivated farmland (NGD) exhibited significantly higher soil organic matter (SOM) and available phosphorus (AP) contents compared to other types. The SOM content (17.36 g/kg) was 56.7% higher than that in bamboo forest (ZL), reaching a statistically significant level (*p* < 0.05). Similarly, the available phosphorus content (32.96 mg/kg) was 85.8% higher than that in abandoned land (LHD), also showing a significant difference. Abandoned land (LHD) showed notable performance in terms of alkali-hydrolyzable nitrogen (AN) and available potassium (AK). Its alkali-hydrolyzable nitrogen content (73.03 mg/kg) was significantly higher than that in bamboo forest by 73.2%, while the available potassium content (115.86 mg/kg) exceeded that in cultivated farmland (NGD) and bamboo forest (ZL) by 49.5% and 45.9%, respectively. In contrast, soil pH and bulk density (SBD) showed no significant differences among the four land use types (A), indicating that these properties were less influenced by land use practices across the four sample sites. Additionally, the soil water content in abandoned land (7.58%) was significantly lower than that in the other three land types, with a reduction ranging from 29.3% to 43.5%, reflecting its weaker water retention capacity. Overall, cultivated farmland tended to accumulate more phosphorus and organic matter, abandoned land had higher nitrogen and potassium nutrient levels, while bamboo forest exhibited relatively lower values across various fertility indicators.

### 3.2. Effect of Different Land Use Types on the Distribution of ASVs in Soil Microbial Communities

**Figure 2:**
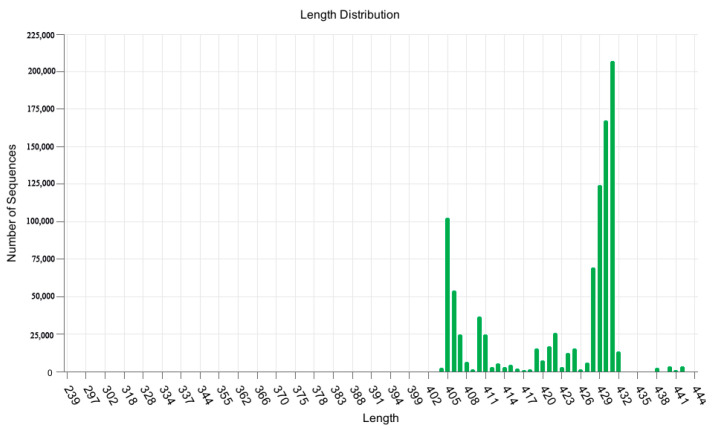
Distribution of soil bacterial sequence lengths in different land use types.

**Figure 3:**
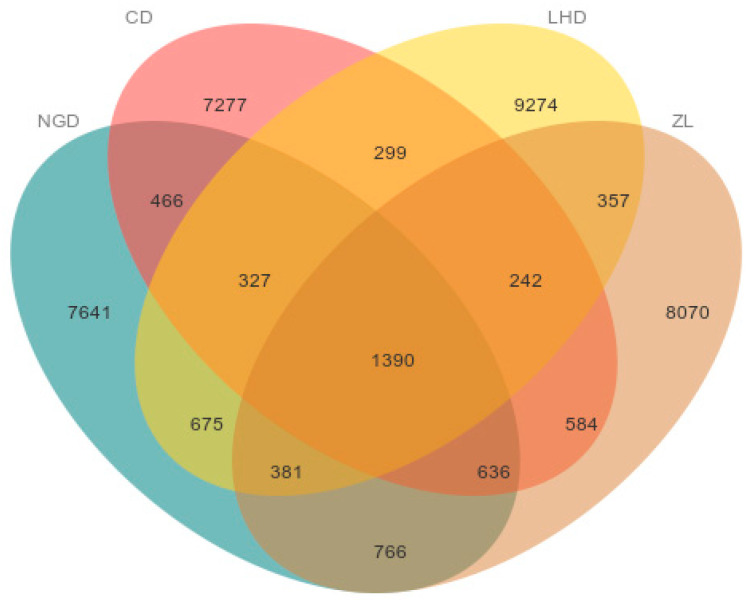
Distribution of soil bacterial ASV counts in different land use types. Abbreviations: NGD: cropland; CD: grassland; LHD: land-holding; ZL: bamboo forest.

Following the removal of low-quality sequences from 1,630,505 raw sequences across four distinct land-use types, a total of 1,461,360 sequences remained. After clustering to eliminate chimeras, 1,098,541 sequences were retained. These sequences exhibited lengths ranging from 239 to 444 base pairs (bp), with the final sequences used for microbial community analysis peaking at 405–430 bp (Figure 2). High-throughput sequencing of the obtained samples was performed using the QIIME2 platform. Analysis of the Venn diagram (Figure 3) revealed that 38,385 operational taxonomic units (ASVs) were detected across the four distinct land use types. Among them, a total of 12,282 ASVs were found in agricultural land, 11,221 ASVs in grassland, 12,945 ASVs in abandoned land, and 12,426 ASVs in bamboo forest. 1390 ASVs were shared by these four different land use types, and the highest number of ASVs was shared between bamboo forest and agricultural land between the two land use types, which amounted to 3173 ASVs. Further analysis shows that 9274 ASVs are unique to the abandoned land, accounting for 24.16% of the total ASVs; followed by bamboo forest with 8070 ASVs, accounting for 21.02%; cropland with 7641 ASVs, accounting for 19.91%; and grassland with 7277 ASVs, accounting for 18.96%.

### 3.3. The Impact of Different Land Use Types on Soil Microbial Community Diversity

In the analysis of alpha diversity within soil bacterial communities, the Shannon index is employed to reflect diversity levels, the Chao 1 index to indicate species richness levels, and the Pielou e-index to reveal the uniformity of soil microbial distribution. Table 2 reveals that no significant differences exist between the four land types in terms of the Chao1 index and Shannon index for soil bacteria (A). However, both indices exhibit their highest values in cultivated land. The Pielou e-index in cultivated land significantly outperforms that of grassland (B), making it the most dominant land type among the four land use categories.

**Figure 4:**
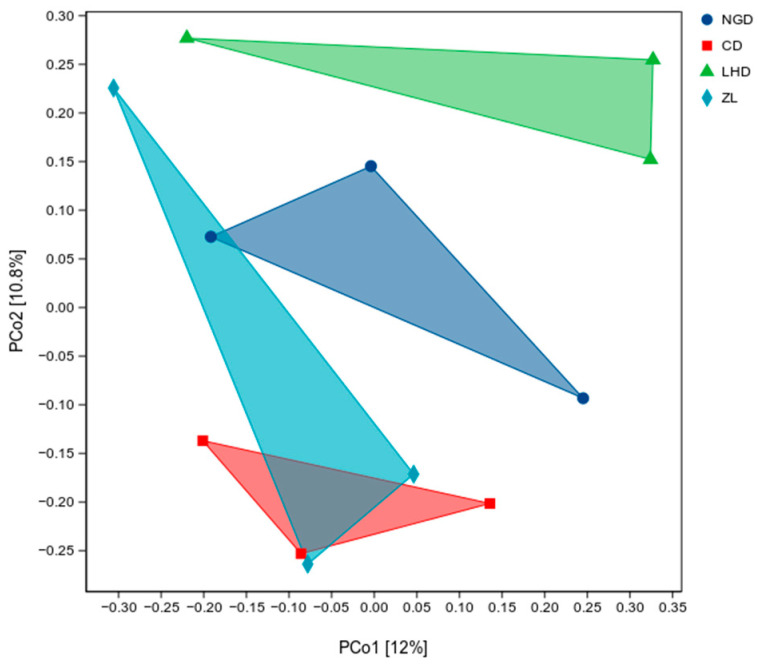
Principal Coordinate Analysis (PCoA) of Bate Diversity in Soil Microbial Communities Across Different Land Use Types. Abbreviations: NGD: cropland; CD: grassland; LHD: land-holding; ZL: bamboo forest.

To investigate the β-diversity of soil microbial communities across four distinct land-use types, a Principal Coordinates of Association (PCoA) analysis was conducted based on the Jaccard distance matrix. The results are shown in Figure 4, where PCo1 and PCo2 explain 12% and 10.8% of the total variation in microbial community composition, respectively. Samples from different groups exhibited distinct separation patterns: those from fallow land (green triangles) clustered predominantly in the upper region of the plot; cultivated land (blue circles) distributed across the upper-middle to lower-middle regions; grassland samples (red squares) concentrated mainly in the lower-middle region; and bamboo forest plots (cyan rhombuses) occupied the lower-middle region towards the left. The polygonal ranges covered by samples from each group show minimal overlap, indicating that soil microbial community composition exhibits significant differences across distinct land use types.

### 3.4. Analysis of Soil Microbial Community Structure in Different Land Use Types

**Figure 5:**
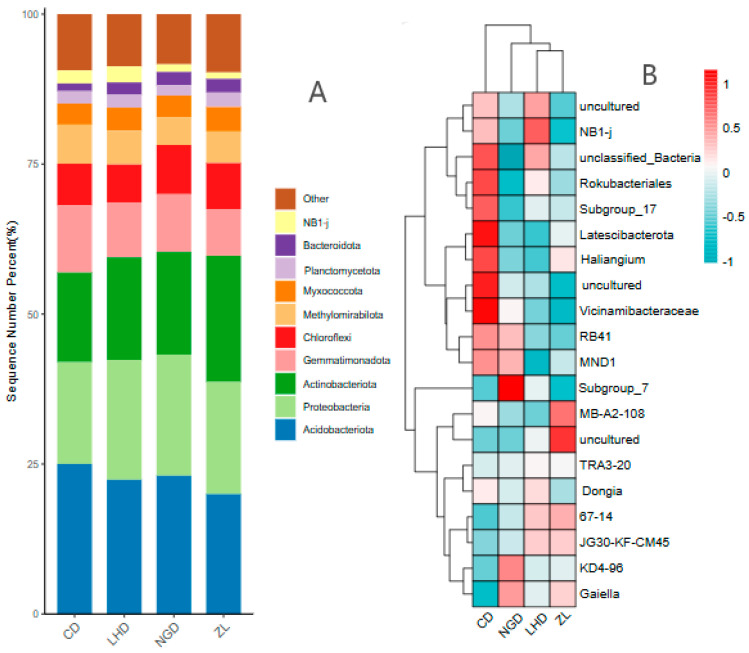
Soil bacterial phylum level (**A**) and genus level (**B**) analysis of different land use types. Abbreviations: CD: grassland; NGD: cropland; LHD: land-holding; ZL: bamboo forest.

By dividing the ASV sequences of soil bacteria from the four land types, there were 43 phyla with 978 genera in bacteria. When soil bacteria are ranked by relative abundance, Figure 5A reveals that the top four phyla among the ten most dominant phyla collectively account for over 67% of all bacterial phyla. Among these, the Acidobacteriota and Gemmatimonadota phyla exhibit the highest abundance in grassland, the Proteobacteria phylum is most prevalent in cultivated land, and the Actinobacteriota phylum dominates in bamboo forests. Acidobacteria ranged from 19.97% to 25.01%, with values of 23.04% ± 3.5% in cultivated land, 25.01% ± 1.02% in grassland, 22.37% ± 4.1% in LHD, and 19.97% ± 0.91% in ZL. Proteobacteria accounted for 16.97% to 20.19%, with values of 20.19% ± 0.35% in farmland, 16.97% ± 1.64% in grassland, 19.96% ± 0.57% in LHD, and 18.7% ± 2.66% in ZL; Actinobacteriota accounted for 14.94–21.06%, with agricultural land at 17.12% ± 1.65%, grassland at 14.94% ± 4.1%, LHD at 17.13% ± 7.25%, and ZL at 21.06% ± 3.19%; Gemmatimonadota accounted for 9.15–11.19%, with values of 9.63% ± 2.04% in cultivated land, 11.19% ± 2.4% in grassland, 9.15% ± 0.11% in LHD, and 7.77% ± 0.92% in ZL.

Through cluster analysis of bacterial genera in soils across four distinct land-use types within the Luo River basin, Figure 5B reveals that the top 20 bacterial genera by relative abundance exhibit overall similarity across different land-use types. However, the strength of correlations among these bacterial genera varies significantly between land-use categories. The overall comparative results reveal that the correlation between the top 20 bacterial genera by relative abundance and grassland utilization type is strongest across the four land-use categories, whereas the correlation with bamboo forest is the weakest. Specifically, within the phylum Acidobacteriota, the genus *Vicinamibacteraceae* exhibits a relatively high relative abundance in grasslands, whereas the genus *Gaiella* demonstrates a comparatively lower relative abundance in grasslands. Within the NB1 phylum, the genus *NB1-j* exhibited the highest relative abundance in fallow land, in stark contrast to the genus *MND1* within the Proteobacteria phylum, which displayed the lowest relative abundance in fallow land. Regarding the Acidobacteriota phylum’s *Subgroup_7* genus, its relative abundance was most prominent in cultivated land, whilst the *unclassified_Bacteria* genus within the unclassified phylum exhibited the lowest relative abundance in cultivated land. Within the Actinobacteriota phylum, *uncultured* bacteria showed the strongest relative abundance in bamboo forests, whereas the *Vicinamibacteraceae* genus exhibited the weakest relative abundance in bamboo forests.

### 3.5. Analysis of Differences in Soil Microbial Community Structure Across Different Land Use Types

**Figure 6:**
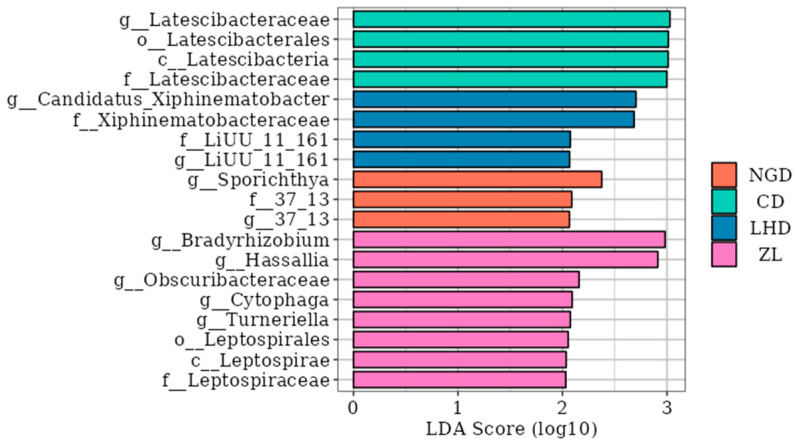
Histogram of LDA values for bacterial species across different land use types. Abbreviations: CD: grassland; NGD: cropland; LHD: land-holding; ZL: bamboo forest.

To further investigate the significantly different species within soil microbial communities across various land use types, analysis was conducted using the LEfSe (LDA Effect Size) method (with the LDA score threshold set at 2). The results, as shown in Figure 6, reveal marked differences in the microbial groups exhibiting significant enrichment characteristics within the rhizosphere soil across different land use types. Among these, bamboo forests (ZL) harbored the highest number of significantly enriched species, with eight significantly different taxa identified. *Bradyrhizobium* exhibited the highest LDA score, indicating it as the most representative biomarker in this habitat. Four significantly different taxa were identified in abandoned farmland (LHD), with *Candidatus_Xiphinematobacter* being the most critical; Three significantly different groups were identified in the cultivated land (NGD), with *Sporichthya* being the most significant indicator species; four important differential species were also identified in the grassland (CD).

### 3.6. Correlation Analysis Between Soil Physical and Chemical Properties and Soil Microbial Community Structure

**Figure 7:**
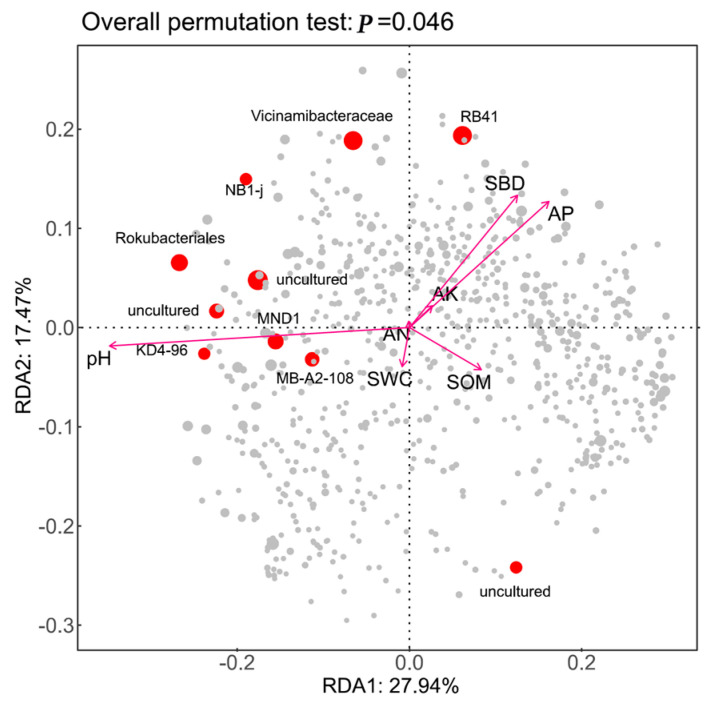
Correlation analysis between soil physicochemical properties and community structure at the genus level of bacteria. Abbreviations: AK, quick-acting potassium; SWC: soil water content; pH: power of hydrogen; SBD: soil bulk density; AP, effective phosphorus; AN: alkali-dissolved nitrogen; SOM, total organic matter.

To analyze the key factors influencing bacterial community changes in soil environments across different land use types, a Redundancy Analysis (RDA) was conducted on soil bacterial community structure and environmental factors including pH, available potassium (AK), available nitrogen (AN), and soil organic matter (SOM). As shown in Figure 7, the first principal component explained 17.47% of the variance, while the second component accounted for 27.94%, with both axes collectively explaining 45.41% of the variance. These components reflect the influence of soil environmental factors on the development of bacterial communities at the genus level. The RDA results (Table 3) indicate that pH (r^2^ = 0.6351, *p* = 0.0040) is the key factor driving differences at the bacterial genus level, while other environmental factors also exert a certain influence on the development of soil bacterial communities at this taxonomic level.

**Figure 8:**
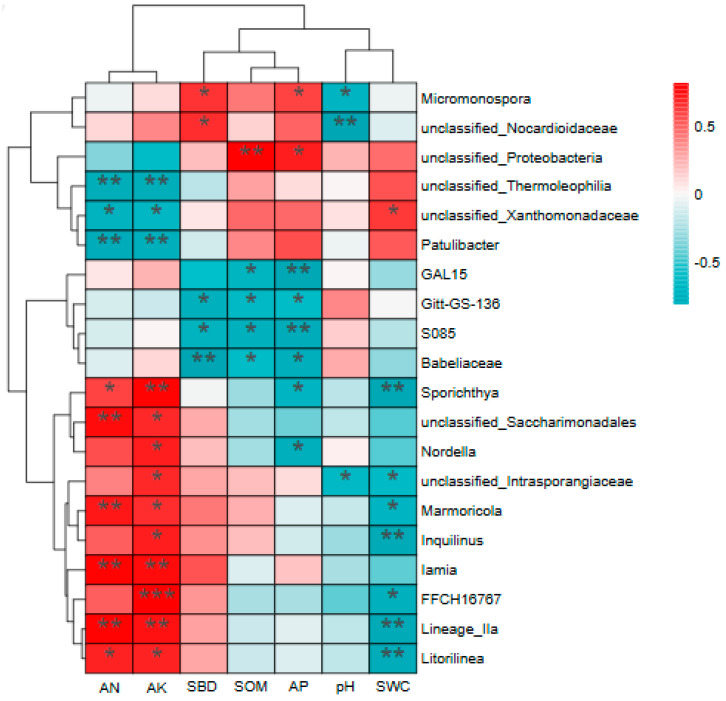
Cluster analysis of soil nutrients and bacterial genera. Abbreviations: SOM, total organic matter; AP, effective phosphorus; AN: alkali-dissolved nitrogen; SBD: soil bulk density; pH: power of hydrogen; AK, immediate potassium; SWC: soil water content. * *p* < 0.05; ** *p* < 0.01; *** *p* < 0.001.

To further investigate the relationship between soil bacterial community composition and soil environmental factors, Spearman’s correlation analysis was conducted between the top 20 bacterial genera and soil physicochemical parameters. As shown in Figure 8, the interactions between bacterial genera and environmental factors were found to be inconsistent. Among these, *Micrococcus*, *unclassified_Nocardiaceae*, *unclassified_Thermophilus*, *Pasteurella*, *Spirobacillus*, *unclassified_Nocillariidae*, *Marmoricola*, and *Iamia* belong to the phylum Actinobacteria; *unclassified_Proteobacteria*, *unclassified_Xanthomonadaceae*, *Nordella*, and *Inquilinus* belong to the phylum Proteobacteria; *Gitt-GS-136*, *S085*, *Litorilinea* belong to the Chlorococcales phylum; *Babeliaceae* belong to the Epibacteroides phylum; *Lineage_Iia* belongs to the Elusimicrobiota phylum; *GAL15* belongs to the GAL15 phylum; *FFCH16767* belongs to the Myxococcota phylum; *unclassified_Saccharimonadales* belong to the Patescibacteria phylum. In the correlation analysis results, among the top 20 bacterial genera by correlation strength, AK exhibited significant differences with 13 bacterial genera, predominantly showing positive significant correlations. The genus FFCH16767 demonstrated a highly significant positive correlation with AK (D). Furthermore, SWC and AP exhibited significant differences with eight bacterial genera, predominantly showing significant negative correlations; AN demonstrated significant differences with nine genera (B); SBD and SOM showed significant differences with five genera (B); and pH exhibited significant differences with three genera (B). *unclassified_Thermoleophilia*, *unclassified_Xanthomonadaceae* and the genus *Patulibacter* exhibited significant negative correlations with AK and AN (B), while *Gitt-GS-136*, *S085* and the genus *Babeliaceae* showed significant negative correlations with SBD, SOM and AP (B).

### 3.7. Analysis of Differences in Soil Microbial Functions Across Land Use Types

The metabolic pathways of soil bacterial communities were analyzed and annotated using PICRUSt2 software and according to the MetaCyc database (Table 4), and the results showed that the metabolic pathways at the soil bacterial level were mainly concentrated in seven categories under different land use types. These are Biosynthesis, Degradation/Utilization/Assimilation, Detoxification, Generation of Precursor Metabolite and Energy, Glycan Pathways, Macromolecule Modification and Metabolic Clusters. Metabolite and Energy, Glycan Pathways, Macromolecule Modification and Metabolic Clusters. Among them, the Biosynthetic Metabolic Pathway is the most dominant metabolic pathway, accounting for 67.99% to 68.84% of the total metabolic pathways.

**Figure 9:**
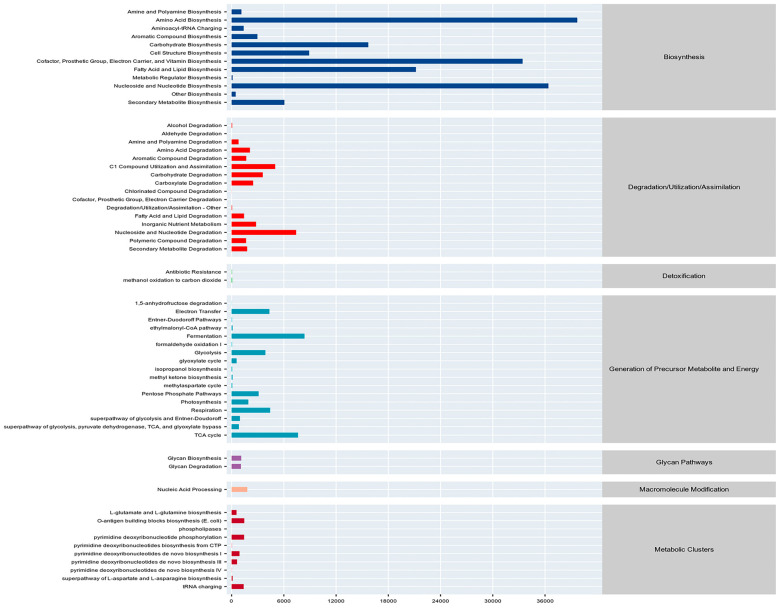
Statistical Analysis of Secondary Metabolic Pathways of Soil Bacteria.

**Figure 10:**
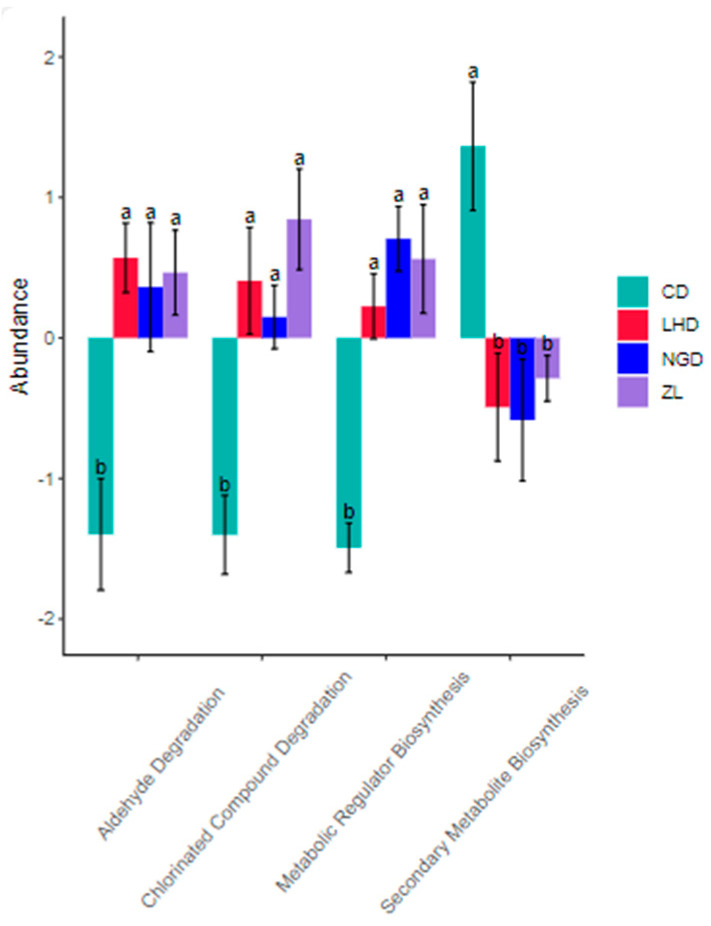
Analysis of differences in secondary metabolic pathways of soil bacteria in different land use types. Abbreviations: CD: grassland; NGD: cropland; LHD: land-holding; ZL: bamboo forest. The letters in the figure indicate significant differences among different land use types (*p* < 0.05).

A total of 60 classes of secondary metabolic pathways were identified through the annotation analysis of secondary metabolic pathways (Figure 9). Further in-depth analysis and comparison of the 60 classes of pathways revealed that there were four classes of secondary metabolic pathways with significant differences (B) between different land use types (Figure 10). These include Secondary Metabolite Biosynthesis and Metabolic Regulator Biosynthesis in the Biosynthesis pathway; Aldehyde Degradation and Chlorinated Compound Degradation in the Degradation/Utilization/Assimilation pathway; and Aldehyde Degradation and Chlorinated Compound Degradation in the Chlorinated Compound pathway. The Degradation/Utilization/Assimilation pathway includes Aldehyde Degradation and Chlorinated Compound Degradation. The grassland land type was significantly better than the other three land types in the Secondary Metabolite Biosynthesis pathway (B), while the remaining three metabolic pathways, abandoned land, agricultural land and bamboo forest, were significantly better than grassland (B).

Among the 60 categories of secondary metabolic pathways, there were 18 categories of metabolic pathways whose relative abundance accounted for more than 1% of the total abundance. Eight categories were attributed to the Biosynthesis pathway, four categories were attributed to the Degradation/Utilization/Assimilation pathway, and six categories were attributed to the Generation of Precursor Metabolite and Energy pathway. These are as follows: Amino Acid Biosynthesis, Aromatic Compound Biosynthesis, Carbohydrate Biosynthesis in the Biosynthesis pathway, Cell Structure Biosynthesis, Cofactor, Prosthetic Group, Electron Carrier, and Vitamin Biosynthesis, Fatty Acid and Lipid Biosynthesis, Nucleoside and Nucleotide Biosynthesis, and Secondary Metabolite Biosynthesis. In the Degradation/Utilization/Assimilation pathway: C1 Compound Utilization and Assimilation, Carbohydrate Degradation, C1 Compound Utilization and Assimilation, Carbohydrate Degradation, Inorganic Nutrient Metabolism, and Nucleoside and Nucleotide Degradation in the C1 Compound Utilization and Assimilation Pathway: Electron Transfer, Fermentation, Glycolysis, Pentose Phosphate Pathways, Respiration, and TCA cycle.

## 4. Discussion

### 4.1. Effects of Different Land Use Types on Soil Physicochemical Properties as Well as Alpha Diversity

Differences in land use practices influence soil physicochemical properties and microbial alpha diversity [38], whilst microbial diversity indices may also reflect the quality of soil structure [39]. In this study, the contents of soil organic matter (SOM) and available phosphorus (AP) in agricultural land were significantly higher (B). This phenomenon may be attributed to the fact that fertilization and tillage management promote the decomposition of organic matter and the cycling of nutrients [40,41,42]. In contrast, the contents of available nitrogen (AN) and available potassium (AK) in abandoned land were significantly higher (B), which is inconsistent with the conclusion from some studies that the nitrogen content in agricultural land is higher [43]. This could be attributed to the minimal human disturbance in abandoned land, which prevents nutrient consumption and loss while facilitating the accumulation and retention of nitrogen and potassium. In the study by Yin [44] et al., it was found that the decomposition of organic matter by microorganisms is also influenced by factors such as the environment and microbial species. Beyond the aforementioned reasons, this may also contribute to the observed differences in nutrient contents across different land use types. In terms of Alpha diversity, the Pielou’s evenness index of agricultural land was significantly higher than that of grassland (B), indicating that agricultural land management practices may promote the evenness of species distribution in the microbial community. However, this study does not consider this a positive phenomenon. This could be because intensive human management, aimed at maximizing economic yields, suppresses naturally dominant species—ultimately leading to simplification and fragility of the ecosystem. While such an ecosystem maintains short-term productivity through external inputs, it comes at the cost of reduced disturbance resistance, compromised ecosystem services, and diminished long-term sustainability. Moderate human disturbance not only helps improve soil structure but also enhances the functional evenness of microorganisms; in contrast, excessive human intervention can cause considerable damage to the soil [45,46].

### 4.2. Effects of Different Land Use Types on Soil Microbial Community Structure

Land use types and changes in the external environment are key factors driving the differentiation of microbial community structures [47,48,49]. In their study, Hu et al. [30] found that different land use patterns not only significantly altered the bacterial community structure but also exerted a certain influence through the response of bacterial communities to environmental factors—which is consistent with the results of this study. In this study, the number of ASVs was the highest in abandoned land and relatively lower in agricultural land. This pattern can be explained as follows: moderate human management may improve soil aeration, but most human disturbances tend to overexploit the soil—an issue avoided in abandoned land due to minimal human interference. The favorable physical and chemical properties of soil in abandoned land facilitate the natural accumulation of microbial diversity and the full differentiation of ecological niches, ultimately leading to a higher number of ASVs. This finding aligns with the study by Sun et al. [50], which revealed that human disturbances exert an impact on soil microbial communities. At the phylum level, the three dominant bacterial phyla in the soil bacterial community of this study were Acidobacteriota, Proteobacteria, and Actinobacteriota. This result is consistent with the findings of Ding et al. [40], which further confirms the core position of these taxa in the soil bacterial community.

In terms of abundance distribution, the three bacterial phyla show significant land use dependence: the abundance of Acidobacteriota is the highest in grassland soils and the lowest in bamboo forest soils; Proteobacteria exhibits a consistent distribution proportion across all tested land use types, demonstrating strong environmental adaptability; Actinobacteriota, on the other hand, has the highest abundance in bamboo forest soils, forming a complementary distribution pattern with Acidobacteriota. This pattern is the result of long-term adaptation between the functions of bacterial phyla and soil microhabitats. In terms of functional correlation, as the core taxon for litter decomposition [51], the high abundance of Acidobacteriota in grasslands can enhance litter degradation and promote soil nutrient accumulation, which explains the relatively high nutrient content in grassland soils. In contrast, the low abundance of this phylum in bamboo forests weakens the decomposition capacity of organic matter and may be one of the key factors leading to the relatively low nutrient content in bamboo forest soils [52]. Actinobacteriota can decompose recalcitrant organic matter and maintain community stability by producing antibiotics [53]. Its high abundance in bamboo forests is not only an adaptive response to the recalcitrant nature of bamboo residues but can also partially compensate for the impact of insufficient Acidobacteriota abundance on nutrient cycling in bamboo forest soils, reflecting the functional compensation effect of the microbial community. The functional redundancy and niche complementarity of these taxa are likely key to maintaining the stability of the soil ecosystem, thus safeguarding soil health. Additionally, they promote plant growth and create a favorable environment for plants. At the genus level, among the top 20 genera with the highest abundance, *Vicinamibacteraceae* and *Latescibacterota* exhibited extremely high abundance in grassland soils. In contrast, the genus *NB1-j* showed dominance in abandoned land soils, while *Subgroup_7* had the relatively highest abundance in agricultural land soils. Additionally, the *uncultured* genus within the phylum Actinobacteriota had the relatively highest abundance in bamboo forest soils.

### 4.3. Influence of Soil Physical and Chemical Properties on Microbial Community Structure

Soil physicochemical factors collectively regulate microbial community structure through resource filtering and habitat filtering [54,55], which is consistent with the study by Wang Shasha et al. [56], who concluded that soil physicochemical factors can affect soil microbial diversity. This study identified soil pH as a key factor influencing community assembly (B), as it directly affects microbial enzyme activity, nutrient availability, and extracellular polymeric substance (EPS) secretion [57,58]. Additionally, an analysis of the correlations between soil physicochemical properties and bacterial genera revealed that available potassium (AK) had significant or highly significant correlations (B, C, D) with 13 bacterial genera among the top 20 most correlated genera. This suggests that potassium availability may play a crucial role in regulating the enrichment and competition of certain bacterial groups (e.g., potassium-solubilizing bacteria or symbiotic bacteria), which aligns with the findings of Zhang Tao et al. [59]. In the regulation of soil microorganisms, besides nitrogen and phosphorus—factors traditionally emphasized—potassium cycling and its functional microbial communities also deserve focused attention. Therefore, in-depth research into the response mechanisms of soil bacterial communities under different land use patterns is of great significance for revealing soil ecological functions and advancing the sustainable use of land.

### 4.4. Effects of Different Land Use Types on Soil Microbial Metabolic Functional Pathways

The prediction results of PICRUSt are of great significance for studying the metabolic functional pathways of soil microorganisms, as they allow the inference of functional information about microbial community structures when direct observation is not feasible [60]. In this study, the Biosynthesis metabolic pathway was identified as the primary first-level metabolic pathway among the seven categories of metabolic pathways in soil bacteria. This pathway is conducive to the survival and development of soil microorganisms, the synthesis of substances, the stability and health of soil ecosystems, as well as the proper cycling of nutrients and transformation of substances [61,62]. In grasslands, the Glycan Pathways exhibited a significant advantage (B) among the four land types. This may be attributed to the abundant plant residues and root exudates in grassland soils [38], which provide rich nutrients for microbial growth and development. In a suitable soil environment, microorganisms engage in mutualistic symbiosis with other microbial taxa; coupled with long-term evolutionary selection of microorganisms, this ultimately enhances the metabolic activity of the Glycan Pathways. Among the four differential secondary metabolic pathways, Secondary Metabolite Biosynthesis showed a significant advantage (B) in grasslands. However, the other three metabolic pathways performed significantly better in abandoned land, agricultural land, and bamboo forests than in grasslands (B). This is similar to the finding by Cheng et al. [63], who observed that different fertilization management levels exert a certain impact on the ecological effects of microorganisms in the soil environment. This suggests that within a specific regional scope, different land use types and management practices do have an influence on microbial metabolic pathways, albeit to a relatively minor extent.

## 5. Conclusions

Within the Luoning section of the Luohe River Basin, different land use types lead to significant differences in soil physicochemical properties and soil microorganisms (B). Grasslands exhibit favorable natural nutrient cycling, minimal disturbance, and good physicochemical properties; moderate human intervention can enhance biodiversity and improve the stability of soil ecosystems. Soil microbial communities are derived from environmental types—under similar environmental conditions, they tend to develop a certain degree of similar structural composition. However, the dominant soil microbial species vary across different land use types. Among the soil bacterial communities of the four land use types, the Biosynthesis pathway was consistently the dominant primary metabolic pathway. Soil pH was identified as the key environmental factor contributing to differences in the community structure of soil bacteria at the genus level across different land use types (B). This study enhances the theoretical understanding of soil–microbe interactions in the Luohe River Basin, providing a reference for the agricultural development, utilization, and management of local soil resources—thereby supporting high-quality agricultural development, optimal utilization of land resources, and sustainable management of the ecological environment. However, this study also has certain limitations, such as the small range of land use types covered and the low explanatory power in RDA analysis. In future research, the study area should be expanded, a more comprehensive set of environmental variables should be adopted, and in-depth analysis should be conducted in combination with process-based models.

## Figures and Tables

**Table 1:** Analysis of soil physicochemical properties of different land use types.

Indexes	NGD	CD	LHD	ZL
pH	7.06 ± 0.14 ^a^	6.97 ± 0.11 ^a^	6.97 ± 0.14 ^a^	6.95 ± 0.08 ^a^
SOM g/kg^−1^	17.36 ± 2.56 ^a^	14.36 ± 0.83 ^ab^	14.60 ± 1.87 ^ab^	11.08 ± 5.30 ^b^
AN mg/kg^−1^	64.46 ± 22.48 ^ab^	60.91 ± 5.94 ^ab^	73.03 ± 4.02 ^a^	42.16 ± 15.47 ^b^
AP mg/kg^−1^	32.96 ± 4.17 ^a^	22.30 ± 8.19 ^ab^	17.74 ± 5.58 ^b^	25.76 ± 7.98 ^ab^
AK mg/kg^−1^	77.46 ± 10.64 ^b^	96.35 ± 15.31 ^ab^	115.86 ± 6.35 ^a^	79.38 ± 17.00 ^b^
SWC/%	13.40 ± 0.75 ^a^	10.72 ± 1.82 ^a^	7.58 ± 1.76 ^b^	11.55 ± 1.25 ^a^
SBD g/cm^3^	1.15 ± 0.12 ^a^	1.01 ± 0.25 ^a^	1.16 ± 0.30 ^a^	0.94 ± 0.10 ^a^

Note: Data are mean ± standard deviation (*n* = 3), different lowercase letters indicate significant differences in indicator values between different land use types (*p* < 0.05), and the maximum mean value is labeled a. NGD stands for farmland, CD stands for grassland, LHD stands for abandoned land, and ZL stands for bamboo forest. Abbreviations: AK, quick-acting potassium; SWC: soil water content; pH: power of hydrogen; SBD: soil bulk density; AP, effective phosphorus; AN: alkali-dissolved nitrogen; SOM, total organic matter.

**Table 2:** Analysis of soil bacterial α-diversity in different land use types.

Land Use Type	Chao1 Exponents	Pielou_e Exponents	Shannon Exponents
NGD	5154.20 ± 663.60 ^a^	0.9139 ± 0.0101 ^a^	11.2219 ± 0.2966 ^a^
CD	4770.29 ± 526.29 ^a^	0.8865 ± 0.0064 ^b^	10.7851 ± 0.1998 ^a^
LHD	5144.56 ± 601.07 ^a^	0.9027 ± 0.0073 ^ab^	11.0919 ± 0.2577 ^a^
ZL	4982.52 ± 589.12 ^a^	0.9071 ± 0.162 ^ab^	11.1026 ± 0.3480 ^a^

Note: Data are mean ± standard deviation (*n* = 3), different lowercase letters indicate significant differences in indicator values between different land use types (*p* < 0.05), and the maximum mean value is labeled a. NGD stands for farmland, CD stands for grassland, LHD stands for abandoned land, and ZL stands for bamboo forest.

**Table 3:** Effects of soil nutrients on the difference of bacterial community level in RDA analysis.

Parameter	r^2^	*p*-Value
pH	0.6351	0.0040
AP	0.3749	0.1164
AK	0.0630	0.7656
AN	0.0116	0.9380
SWC	0.0732	0.7356
SBD	0.3334	0.1509
SOM	0.1705	0.4108

Abbreviations: AK, quick-acting potassium; SWC: soil water content; pH: power of hydrogen; SBD: soil bulk density; AP, effective phosphorus; AN: alkali-dissolved nitrogen; SOM, total organic matter.

**Table 4:** Statistics of soil bacterial primary metabolic pathways in different land use types.

Metabolic Pathways	NGD	CD	LHD	ZL
Biosynthesis	0.6799 ± 0.0089 ^a^	0.6884 ± 0.0060 ^a^	0.6817 ± 0.0155 ^a^	0.6800 ± 0.0086 ^a^
Degradation/Utilization/Assimilation	0.1280 ± 0.0089 ^a^	0.1202 ± 0.0049 ^a^	0.1276 ± 0.0135 ^a^	0.1278 ± 0.0074 ^a^
Detoxification	0.0005 ± 0.0002 ^a^	0.0003 ± 0.00004 ^a^	0.0004 ± 0.0003 ^a^	0.0004 ± 0.0002 ^a^
Generation of Precursor Metabolite and Energy	0.1489 ± 0.0010 ^a^	0.1476 ± 0.0012 ^a^	0.1480 ± 0.0028 ^a^	0.1492 ± 0.0024 ^a^
Glycan Pathways	0.0088 ± 0.0001 ^bc^	0.0090 ± 0.0002 ^a^	0.0086 ± 0.00003 ^c^	0.0089 ± 0.0001 ^ab^
Macromolecule Modification	0.0073 ± 0.0004 ^a^	0.0076 ± 0.0004 ^a^	0.0074 ± 0.0005 ^a^	0.0071 ± 0.0002 ^a^
Metabolic Clusters	0.0266 ± 0.0007 ^a^	0.0269 ± 0.0001 ^a^	0.0263 ± 0.0006 ^a^	0.0267 ± 0.0009 ^a^

Note: Data are mean ± standard deviation (*n* = 3), different lowercase letters indicate significant differences in indicator values between different land use types (*p* < 0.05), and the maximum mean value is labeled a. NGD stands for farmland, CD stands for grassland, LHD stands for abandoned land, and ZL stands for bamboo forest.

## Data Availability

The microbial data have been uploaded to the NCBI database, and the serial number is BioProject: PRJNA1264484. Other data can be provided upon request.
